# Raman Spectroscopy of Head and Neck Cancer: Separation of Malignant and Healthy Tissue Using Signatures Outside the “Fingerprint” Region

**DOI:** 10.3390/bios7020020

**Published:** 2017-05-14

**Authors:** Stephen Holler, Elaina Mansley, Christopher Mazzeo, Michael J. Donovan, Maximiliano Sobrero, Brett A. Miles

**Affiliations:** 1 Department of Physics & Engineering Physics, Fordham University, 441 East Fordham Road, Bronx, NY 10458, USA; emansley@fordham.edu (E.M.); cmazzeo2@fordham.edu (C.M.); 2 Icahn School of Medicine at Mount Sinai Hospital, 1 Gustave L. Levy Place, New York, NY 10029, USA; michael.donovan@mssm.edu (M.J.D.); maximiliano.sobrero@icahn.mssm.edu (M.S.); brett.miles@mountsinai.org (B.A.M.)

**Keywords:** Raman spectroscopy, head and neck cancer, tissue diagnostics, principal component analysis, multivariate statistics, otolaryngology

## Abstract

The ability to rapidly and accurately discriminate between healthy and malignant tissue offers surgeons a tool for in vivo analysis that would potentially reduce operating time, facilitate quicker recovery, and improve patient outcomes. To this end, we investigate discrimination between diseased tissue and adjacent healthy controls from patients with head and neck cancer using near-infrared Raman spectroscopy. Our results indicate previously unreported peaks in the Raman spectra that lie outside the conventional “fingerprint” region (400 ${\mathrm{cm}}^{-1}$–1800 ${\mathrm{cm}}^{-1}$) played an important role in our analysis and in discriminating between the tissue classes. Preliminary multivariate statistical analyses of the Raman spectra indicate that discrimination between diseased and healthy tissue is possible based on these peaks.

## 1. Introduction

Head and neck squamous cell carcinoma is currently ranked as the 6th most common cancer in the world. There are an estimated 650,000 incident cases and over 300,000 resultant deaths from head and neck squamous cell carcinoma (SCC) annually [1]. Human Papillomavirus (HPV) has increasingly been recognized as a major risk factor in this type of cancer, especially in oropharyngeal squamous cell carcinoma. Two high-risk strains of HPV (16,18) are oncogenic and are responsible for 12.8%–59.9% of head and neck squamous cell carcinomas [2,3]. Removal of malignant tissue requires a careful balance of options: remove too much and it may negatively impact the patient’s quality of life, or remove too little and the cancer will return. Therefore, accurate identification of cancer margins, ideally in vivo, is crucial to achieving this balance and optimizing treatment. Currently, a pathologist is required to determine whether or not the surgical margins are positive or negative, which takes a significant amount of time.

In an effort to improve margin detection and patient outcomes optical diagnostics are becoming more widely used [4]. Spectroscopic techniques such as fluorescence [5,6] and Raman spectroscopy [7,8,9,10] have emerged as potent cancer diagnostic tools. Raman spectroscopy, a label-free approach, does not require special dyes or other, potentially toxic, probes (e.g., quantum dots and carbon nanotubes [11]) to elicit signal. Although Raman scattering signals can be weak, it does offer molecular specific detection in the vicinity of the probe.

Raman spectroscopy has been used extensively as a cancer diagnostic tool for ex vivo and minimally invasive in vivo analyses of affected tissues for more than two decades [12]. Despite the wide-spread applicability of Raman spectroscopy, our focus is specifically on cancers of the head and neck. Improvements and innovations in instrumentation and computational approaches for discrimination coupled with the growing incidence of head and neck cancers has resulted in numerous studies in recent years [13,14,15,16,17,18]. Much of this work has focussed on the conventional fingerprint region (400–1800 ${\mathrm{cm}}^{-1}$), corresponding to biochemically relevant spectral signatures, although there have been efforts that have examined higher wavenumber Raman shifts (>2000 ${\mathrm{cm}}^{-1}$) [19] for additional discriminatory signatures. In particular, the high wavenumber Raman shifts offer reduced autofluorescence background signals while providing additional biochemical signatures useful in tissue characterization [20]. Multivariate analyses have proven adept at enhancing spectral differences allowing for good separation among healthy and diseased tissue classes. However, the complexity of classification may be confounded by patient factors, such as tobacco and alcohol use, which can lead to variations in the observed spectra.

We have been investigating optical techniques that may be used for in vivo identification of cancer margins [21]. The present work analyzes the Raman signatures from tissue samples (healthy and diseased) from patients who have undergone treatment at Mount Sinai Hospital in New York City. The Raman spectra were analyzed using principal component analysis in order to demonstrate discrimination between the cancerous samples and the healthy controls. Results from this study indicate that although the majority of the spectral signatures are nearly identical in our cancerous tissue and healthy controls, there are several peaks, including *several previously unreported peaks* in the 130–400 ${\mathrm{cm}}^{-1}$ Raman shift region, that proved useful in differentiating between the healthy and malignant samples. In addition, we observe systematic intensity differences among the spectra, where the intensity of the Raman signal (peaks + baseline) in the cancerous samples are identifiably higher than their corresponding control. In what follows, we will introduce the experimental arrangement (Section 2), briefly discuss the multivariate analysis (Section 3), and present our results (Section 4).

## 2. Experimental Setup

### 2.1. Tissue Samples

Tissue samples from head and neck tumors (including the tonsils) were excised from several patients along with healthy controls. Control tissue is similar to mucosa sampled from the oral cavity but not immediately adjacent to the tumor due to possible field effect. The patient data, summarized in Table 1, represents four different patients and sixty different measurements on different tissue samples. All the patients had some history of tobacco use. Each patient had several tissue IDs, each of which corresponded to at least five different samples. The tissue samples were collected for scientific use according to Institutional Review Board procedures at Mt. Sinai Hospital and confidentiality of the patients was maintained in compliance with federal HIPAA regulations.

Slices between 200 μm and 500 μm thick of these tissue samples were prepared on microscope slides for analysis. The mean dimension of the tissues studied were 600 μm × 500 μm × 400 μm [22]. Some of the tissue samples were treated with formalin fixed hematoxylin and eosin stain (H & E stain). While fixation and staining does alter the chemical composition of the tissue, this did not appear to affect the spectroscopic discrimination capability, consistent with recent findings [23], or our multivariate analysis. The tissues were stored at −${80\;}^{\circ }$C long term and prior to the experiments were kept at −${20\;}^{\circ }$C. During the actual measurements, the tissues were allowed to thermalize to room temperature prior to performing any measurements. Table 1 summarizes the tissue samples used in this study. In all, Raman spectra from sixty different tissues samples were acquired and analyzed. These tissue samples were distributed among the different patients with several samples from each patient.

### 2.2. Apparatus

**Figure 1:**
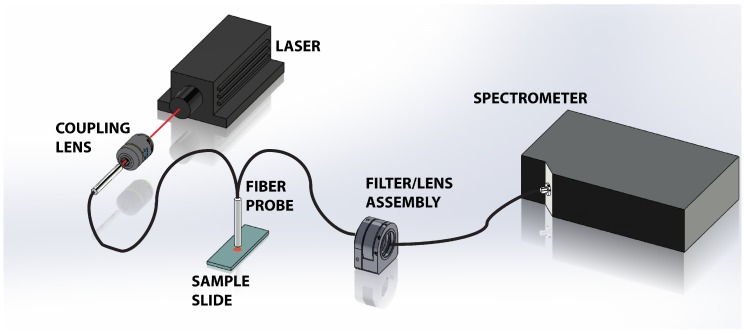
Schematic of the experimental setup showing the 785 nm laser directed into the Raman probe via the 10× objective lens. The probe illuminates the tissue sample and collects the scattered light. The elastically scattered signal is removed via a long pass filter in the filter/lens assembly before the light is transmitted into the Maya Pro 2000 NIR spectrometer for dispersion and storage.

Figure 1 shows a schematic of the experimental apparatus. A 300 mW, 785 nm near-infrared laser (LaserGlow, Toronto, ON, Canada) is used to illuminate the tissue sample. To prevent saturation of the spectrometer with strong residual Rayleigh signal, the beam was attenuated and coupled into a multimode optical fiber reflection probe (Ocean Optics, Dunedin, FL, USA) through a 10× objective (coupling lens, ThorLabs, Newton, NJ, USA). The attenuation and coupling losses resulted in approximately 1 mW of power emerging from the fiber probe. The probe, comprised of a read out core surrounded by a ring of 200 μm diameter core illumination fiber, was positioned approximately 1 mm above the sample. The divergence of the light emanating from the probe illuminates the tissue sample with a spot that is approximately 700 μm on the tissue, which is a significant fraction of the tissues under study. This results in spatial averaging of the Raman scattering signal collected by the probe. The scattered light is collected via the central read fiber and is transmitted through the second leg of the probe to the filter assembly. The emerging light enters the filter/lens assembly where it is collimated and passes through a 785 nm Raman edge filter (Edmund Optics, Barrington, NJ, USA) which is designed to block the laser and elastic scattering while transmitting light greater than 792.5 nm. The filtered light is then focused back into an optical fiber and directed to a Maya Pro 2000 NIR spectrometer (Ocean Optics). The light is then dispersed across the detector and recorded to a computer for further analysis.

The tissue samples were illuminated by the 785 nm laser and data was collected for a period of 17 s. For each tissue sample, this was repeated 20 times in order to reduce “background noise” and integrate the weak Raman signals. A background spectrum from a blank microscope slide was also recorded under the same conditions. The raw spectroscopic data was exported to MatLab, where the spectra were processed to remove the background signals, and perform the multivariate analysis.

## 3. Principal Component Analysis

The measured Raman spectra were used as inputs for a Principal Component Analysis (PCA) algorithm. For our analysis we employed routines that are contained within MatLab’s Statistics Toolbox. The use of these algorithms provided us flexibility in exploring the efficacy of the discrimination scheme on this preliminary data.

In essence, PCA serves to reduce the dimensionality of the data set by formulating an orthogonal basis set where the first few principal components contain the majority of the information in the data set [24]. In effect the principal components (PCs) extract the most pertinent spectral information that can be used to reconstruct the original spectrum. In doing so, the dimensionality of the data **X** (M × N) is reduced and may be expressed as

(1) $$X=\sum_{n=1}^{A}t_{n}⊗p_{n}+E=T\cdot P+E$$

where the limit *A* is taken to be the number of retained PCs, **T** (M × A) is the scores matrix and **P** (A × N) is the loadings matrix, and **E** is the error matrix. The principal components were computed using the princomp function in MatLab. In general, for discrimination purposes, we retain those principal components that contained 99% of the information content.

## 4. Results

**Figure 2:**
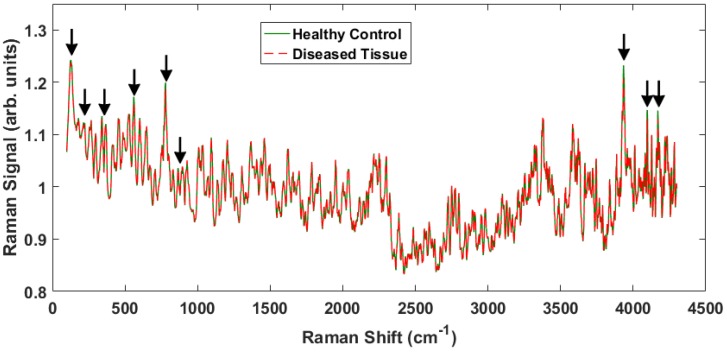
Representative Raman spectra from two different samples, one healthy and one cancerous over the entire ∼4000 ${\mathrm{cm}}^{-1}$ Raman shift signal. The peaks that show differences between the healthy tissue and the cancerous tissue are indicated by the arrows.

The results from this study indicate that one can readily differentiate between cancerous and noncancerous tissue samples by analyzing the spectroscopic data obtained through Raman spectroscopy. Figure 2 shows representative spectra from healthy and malignant tissue obtained with the apparatus (Figure 1) taken across many days and corroborated with different probe configurations. The probe employed for these experiments collected a small fraction of the scattering producing weak signals that required some post-processing. A 5 point smoothing function was applied to the data shown as well as a normalization to the 1000 ${\mathrm{cm}}^{-1}$ raw value. The signal from our samples was nearly the same over the measured 4300 ${\mathrm{cm}}^{-1}$.

There are some notable differences in the two spectra that enable discrimination between the healthy and malignant tissue samples. The Raman peaks that played a key role in separating the tissue classes are marked with arrows in Figure 2. Some of the marked peaks have been identified in the literature. Notably, within the conventional fingerprint region (400 ${\mathrm{cm}}^{-1}$–1800 ${\mathrm{cm}}^{-1}$), the band at 500 ${\mathrm{cm}}^{-1}$ is associated with glycogen, and the peak at 780 ${\mathrm{cm}}^{-1}$ is due to nucleic acids, specifically the nucleobase adenine [8,14,25,26]. Note that the specific locations of the Raman shifts will vary slightly depending on the local environment (e.g., water content). The high frequency and low frequency peaks have not been identified, but are actually observed to undergo larger intensity changes and therefore provide greater discriminatory capability.

**Figure 3:**
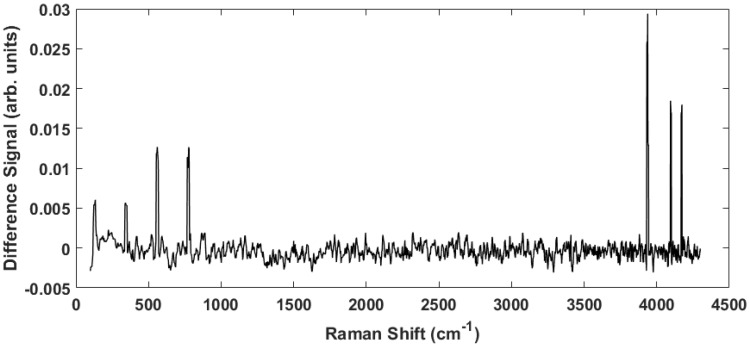
The difference spectrum obtained by subtracting the cancerous spectrum from the healthy spectrum in Figure 2. This spectrum highlights the peaks indicated above.

To accentuate the variations in the observed Raman spectra from the different tissue sample classes, a representative difference spectrum was generated from the two spectra presented in Figure 2. Figure 3 shows the result. There is a general suppression across the entire Raman shift spectrum (i.e., the normalized healthy tissue samples show slightly greater intensity across the spectrum consistent with previous measurements on adenocarcinoma [27]), but more importantly the differences of the discriminatory peaks are more noticeable. The two distinct lower frequency peaks at 130 ${\mathrm{cm}}^{-1}$ and 350 ${\mathrm{cm}}^{-1}$ as well as a broad peak near 200 ${\mathrm{cm}}^{-1}$ are of interest because they are previously unreported, lie close to the fingerprint region, and proved useful in our statistical analysis. We will return to these peaks later. In addition, smaller, less distinctive differences are observed at 870 ${\mathrm{cm}}^{-1}$ and 940 ${\mathrm{cm}}^{-1}$, which have been previously reported as arising from Tryptophan and C–C stretching, respectively [28].

**Figure 4:**
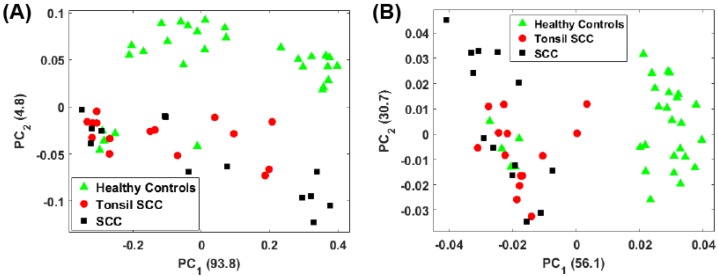
Plot of the first two principal component scores for (**A**) for the full spectrum (100 ${\mathrm{cm}}^{-1}$–4300 ${\mathrm{cm}}^{-1}$) and (**B**) the conventional fingerprint (400 ${\mathrm{cm}}^{-1}$–1800 ${\mathrm{cm}}^{-1}$). Both plots show good separation between the healthy controls and the malignant tissue samples (both tonsil squamous cell carcinoma and squamous cell carcinoma). The numbers in parentheses represent the information content associated with each principal component.

Figure 4A shows a scatter plot of the first two principal component scores for the Raman spectra obtained from the patients listed in Table 1 using the full spectral signature. The PCA results show separation among the healthy and diseased classes. The diseased tissue class is comprised of two sub-classes: squamous cell carcinoma (SCC) and SCC specifically from tonsil tissue. These classes are represented in Figure 4 as green triangles (healthy controls), SCC (black squares), and tonsil SCC (red circles). One can see how there is a distinct differentiation between the location of the cancerous samples and their adjacent controls on this scatter plot, indicating that one can differentiate cancerous samples from non-cancerous samples by analyzing the PC scores of the spectroscopic data. For comparison we also plot the first two PC scores using a subset of the spectral data constrained within the conventional fingerprint region (Figure 4B). The same convention is used for labeling the classes. The results from an analysis of this region also show good separation of the healthy and diseased tissue classes.

For the analysis of both the full spectrum and conventional fingerprint spectrum, shown in Figure 4, there are five data points from healthy tissue resected from patient 5120 (Table 1) that have been associated with the diseased tissue class. The remaining tissue samples from patient 5120 showed clear distinction between the healthy and diseased tissue. We suspect this particular tissue sample, despite being labeled a healthy control, is in fact diseased, or at least in the early stages of disease, though we cannot rule out mislabeling.

**Figure 5:**
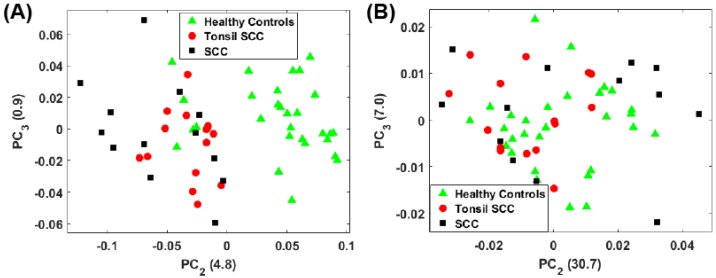
Plot of the second and third principal component scores for (**A**) for the full spectrum (100 ${\mathrm{cm}}^{-1}$–4300 ${\mathrm{cm}}^{-1}$) and (**B**) the conventional fingerprint (400 ${\mathrm{cm}}^{-1}$–1800 ${\mathrm{cm}}^{-1}$). The full spectrum analysis reveals a distinct boundary between the healthy and diseased tissue, however no obvious separation of the data is observed when looking at the conventional fingerprint region. This increased separation is due to the peaks observed outside the conventional fingerprint regime. The numbers in parentheses represent the information content associated with each principal component.

For the full spectral analysis the first PC represents most of the spectral information. This appears to be the result of a strong Rayleigh signature in the low Raman shift region. Because of this, we also plot the second and third PC scores for the full spectrum (Figure 5A) and the conventional fingerprint region (Figure 5B). These classes are again represented as green triangles (healthy controls), SCC (black squares), and tonsil SCC (red circles). Notably, the full spectrum analysis still shows a clear separation among the classes of healthy and diseased tissue. The same five data points from patient 5120 are readily seen to be associated with the diseased class in Figure 5A. However, when only the conventional fingerprint region is analyzed using PCs 2 and 3, no clear distinction is observed among the tissue classes.

**Figure 6:**
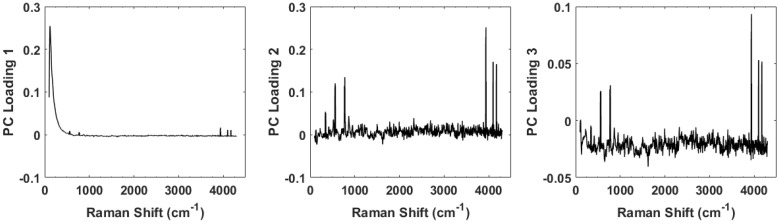
Plots of the loadings for the first three principal components. The loadings for the first is dominated by a large Rayleigh peak near the 0 ${\mathrm{cm}}^{-1}$ shift, but small peaks can be seen further out. The loadings for the second and third principal components clearly show the peaks and contribute strongly to the discrimination capability of the spectra.

The separation observed in the full spectrum analysis has significant contributions from the peaks identified in the difference spectrum (Figure 3). This can be seen by examining the principal component loadings. Figure 6 shows plots of the loadings for the first three principal components. Evident in all three plots are the peaks observed in Figure 3 whose strength differs among the healthy controls and the diseased tissue samples. While the loadings for the first PC is dominated by a large Rayleigh signal, the signature of these peaks is evident. We are currently engaged in improving the apparatus to eliminate any stray light entering the spectrometer and minimizing the Rayleigh peak.

**Figure 7:**
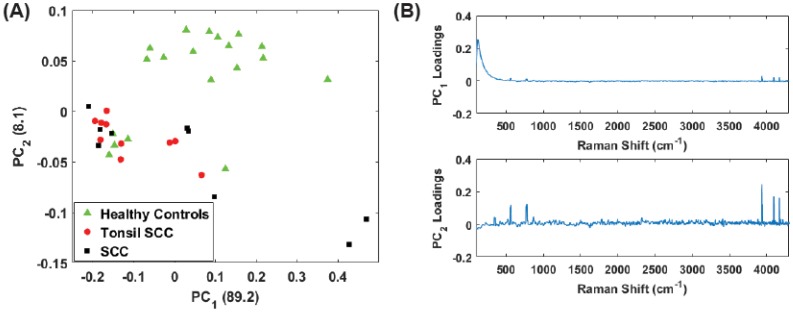
(**A**) Plot of the first two principal component (PC) scores for only the unstained tissue samples. The analysis was performed on the full spectral data, and show good separation between healthy and diseased tissue. The numbers in parentheses represent the information content associated with each principal component. (**B**) Corresponding loadings plots for the PCs shown in (**A**).

A check of our results was performed to verify that the H & E stain was not impacting the spectroscopic discrimination of our results since the analysis shown in both Figure 4 and Figure 5 utilized both stained and unstained samples to increase the size of the data set. We were confident that we could analyze the mixed samples because H & E stain has low absorption at our operating wavelength (785 nm) and therefore should produce little fluorescence to interfere with the measured Raman spectra [29,30]. Furthermore, Andronie, et al. concluded “that hematoxylin and eosin does not interfere in the tissue Raman signal” further bolstering our hypothesis that diagnostic information is still available [23]. Performing the same preprocessing and PC analysis on the full spectrum with only the unstained samples resulted in the scores plots shown in Figure 7A. The associated loadings are seen in Figure 7B. As can be seen from these results, even though staining and fixation do alter the chemical composition of the tissue, clear separation among the healthy controls and diseased tissue samples is observed.

**Figure 8:**
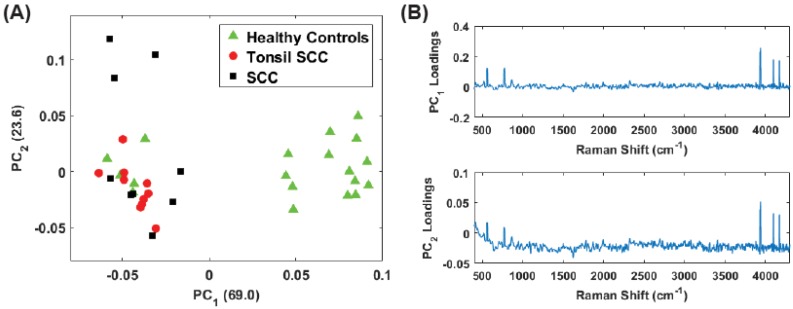
(**A**) Plot of the first two PC scores for only the unstained tissue samples. The analysis was performed in the Raman shift region from 400 ${\mathrm{cm}}^{-1}$–4300 ${\mathrm{cm}}^{-1}$ and show good separation between the healthy and diseased tissue classes. The numbers in parentheses represent the information content associated with each principal component. (**B**) Corresponding loadings plots for the PCs shown in (**A**).

**Figure 9:**
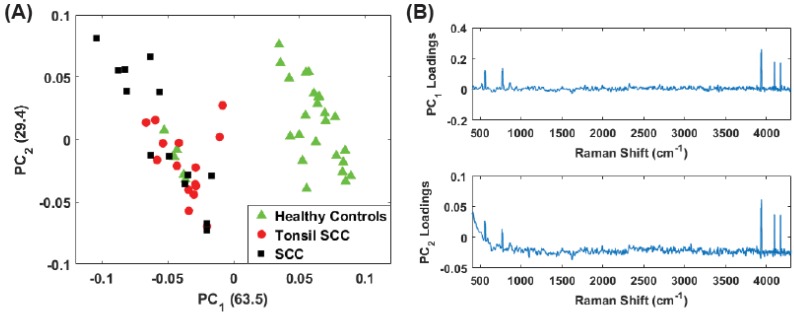
(**A**) Plot of the first two PC scores for only the unstained tissue samples. The analysis was performed in the Raman shift region from 400 ${\mathrm{cm}}^{-1}$–4300 ${\mathrm{cm}}^{-1}$ and show good separation between the healthy and diseased tissue classes. The numbers in parentheses represent the information content associated with each principal component. (**B**) Corresponding loadings plots for the PCs shown in (**A**).

While the low frequency peaks appear to have diagnostic capability, we chose to reanalyze the data due to the presence of the Rayleigh peak that dominates ${\mathrm{PC}}_{1}$. Eliminating the low frequency region (<400 ${\mathrm{cm}}^{-1}$) we performed the same principal component analysis on the full data set (stained and unstained samples) as well as only the unstained samples in the region from 400 ${\mathrm{cm}}^{-1}$ to 4300 ${\mathrm{cm}}^{-1}$. This removes a significant portion of the Rayleigh peak while retaining the peaks observed in the 3500 ${\mathrm{cm}}^{-1}$–4300 ${\mathrm{cm}}^{-1}$ region. Figure 8 shows the results for the unstained tissue samples. The scores for PCs 1 and 2 are shown in Figure 8A and the corresponding loadings plots are shown in Figure 8B. As before, the healthy and diseased tissue show a clear separation. In the loadings for ${\mathrm{PC}}_{2}$, a weak tail of the Rayleigh signal can be seen, but this appears to have a smaller role in this latest analysis compared to earlier. Figure 9 shows the results for all the data (stained and unstained). The scores for PCs 1 and 2 are shown in Figure 9A and the corresponding loadings plots are shown in Figure 9B. The separation of healthy and diseased tissue is still apparent, however, along the ${\mathrm{PC}}_{1}$ axis, the distance between the classes appears narrower. This is likely due to the inclusion of the stained tissue samples and consequent chemical alteration due to staining and fixation. The same weak tail of the Rayleigh signal can be seen be seen in the loadings for ${\mathrm{PC}}_{2}$, but does not appear to affect the outcome.

**Figure 10:**
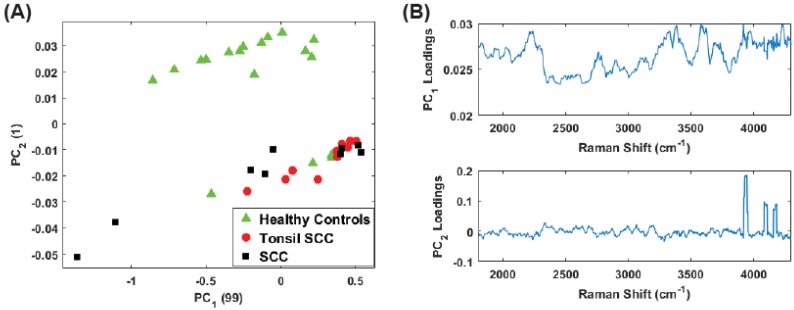
(**A**) Plot of the first two PC scores for the unstained tissue samples. The analysis was performed in the Raman shift region from 400 ${\mathrm{cm}}^{-1}$–4300 ${\mathrm{cm}}^{-1}$ and show good separation between the healthy and diseased tissue classes. The numbers in parentheses represent the information content associated with each principal component. (**B**) Corresponding loadings plots for the PCs shown in (**A**).

**Figure 11:**
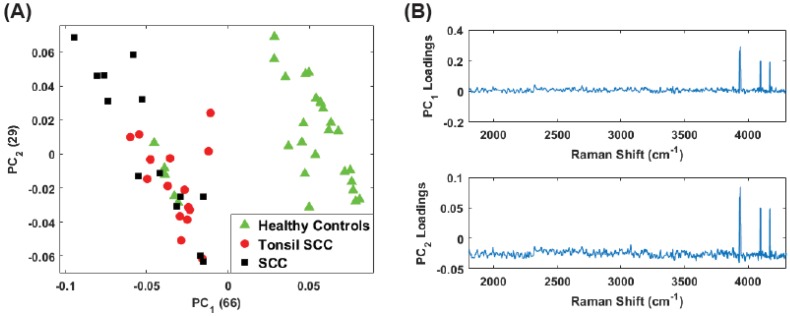
(**A**) Plot of the first two PC scores for all (stained and unstained) the tissue samples. The analysis was performed in the Raman shift region from 1800 ${\mathrm{cm}}^{-1}$–4300 ${\mathrm{cm}}^{-1}$ and continues to show good separation between the healthy and diseased tissue classes despite the inclusion of the stained tissue samples. The numbers in parentheses represent the information content associated with each principal component. (**B**) Corresponding loadings plots for the PCs shown in (**A**).

Finally, we analyzed only the high frequency shift regime (1800 ${\mathrm{cm}}^{-1}$–4300 ${\mathrm{cm}}^{-1}$) for the unstained samples. Figure 10A shows the scores plot while Figure 10 plots the corresponding loadings. Here the vast majority of the information is contained in ${\mathrm{PC}}_{1}$. Despite this, the separation among the diseased and healthy tissue is observed. However, it is noteworthy that the discrimination is largely a consequence of ${\mathrm{PC}}_{2}$. From the loadings plot, some small peaks are observed for ${\mathrm{PC}}_{1}$ but the ${\mathrm{PC}}_{2}$ loadings are dominated by the peaks in the 3500 ${\mathrm{cm}}^{-1}$–4300 ${\mathrm{cm}}^{-1}$ region, i.e., those high frequency peaks noted in the difference spectrum (Figure 3, thus supporting our contention of the discriminating capability of these peaks. The same analysis was repeated on the data set including all (stained and unstained) tissue samples. The scores plot is shown in Figure 11A. The corresponding loadings are shown in Figure 11B and are dominated by the high frequency Raman shift peak in the region from 3500 ${\mathrm{cm}}^{-1}$–4300 ${\mathrm{cm}}^{-1}$. The separation between the diseased and healthy tissue classes in clear although the data is reoriented from the unstained case (Figure 10) because both PCs have loadings that weight the high frequency peaks. Regardless, the mixing of stained and unstained tissue samples has not impacted the diagnostic capability despite any chemical alterations to the tissue due to staining and fixation.

In all cases, the same five data points addressed earlier score among the diseased tissue; however, our contention is that this sample was mislabeled. As we anticipated, the results of our analysis of only the unstained tissue samples are consistent with those presented early for the data set comprised of both stained and unstained samples.

## 5. Summary and Conclusions

We reported on the use of Raman spectroscopy at 785 nm to discriminate between healthy and cancerous tissue samples from patients with head and neck cancer, and the role that previously unreported signatures played. The observed spectral signatures were processed with a principal component algorithm to yield a clear delineation between the tissue classes. The discrimination was dominated by several previously unreported peaks in both low and high frequency regions. The low frequency peaks are of particular interest because their proximity to the fingerprint region.

The observed Raman spectra demonstrate distinct and previously unreported peaks in the low frequency shift signal region (130–400 ${\mathrm{cm}}^{-1}$) which lies outside the conventional “fingerprint” region (400 ${\mathrm{cm}}^{-1}$–1800 ${\mathrm{cm}}^{-1}$) but were important to discriminating between the cancerous and healthy tissue samples. The precise origin of these low frequency shifts remains unknown. While the 130 ${\mathrm{cm}}^{-1}$ peak lies close to the cut-on wavelength of the filter it was observed to change between healthy and diseased tissue in numerous measurements. The observed changes seem to be indicative of a change within the tissue sample as it transitions from a healthy to a cancerous state. Low-frequency Raman shift signatures are typically associated with crystalline structures and are used in the pharmaceutical industry for dosage analysis [31]. Morphological vibrations of whole virus have been of interest to researchers and also result in low-frequency Raman shifts [32,33,34], however these shifts are extremely low and generally fall outside the region of our observations. While these peaks are not likely due to the HPV virus itself, the differences in the observed peaks could be indicative of the integration of viral DNA into the cell and the expression of oncogenes. One possible explanation for these peaks is that they correspond to nucleic acids, however, such a designation requires further study.

The observed signature in the 130 ${\mathrm{cm}}^{-1}$ to 400 ${\mathrm{cm}}^{-1}$ range is likely due to the changes the cells in the tissue sample undergo as the cancer develops. Measurements of both healthy and diseased cervical tissue show similar degradation of signal, particularly the glycogen signature, as the tissue becomes cancerous [25]. Although the glycogen signature lies within the conventional fingerprint window and did play a role in this discrimination, there is no reason to believe that such changes do not also occur at lower Raman shift frequencies as other glyco- compounds do present in this region [35].

Further study of this new spectral signature for the differentiation of healthy and malignant tissue is ongoing. Despite its unknown origin, the spectral differences between the tissues samples is sufficiently clear that multivariate statistical analyses demonstrate excellent discrimination capability, and offers the promise of a real-time tool for in vivo diagnostics which may ultimately improve surgical outcome and the patient’s quality of life.

## Figures and Tables

**Table 1:** Summary of the patient data and tissue type for the samples used in this study.

Patient	Tissue ID	Sex/Age	Diagnosis
005094	S01A	F/53	Control
005094	S01B	F/53	Tonsil SCC
005112	S01A	M/49	Tonsil SCC
005118	S01A	M/49	Tonsil SCC
005120	S01A	M/70	Control
005120	S01B	M/70	Control
005120	S01C	M/70	SCC
005120	S01D	M/70	SCC
005120	S01E	M/70	SCC
